# Interaction between 24 h Urinary Free Cortisol and Obesity in Hypertension-Mediated Organ Damage in Patients with Untreated Hypertension

**DOI:** 10.31083/RCM25598

**Published:** 2025-01-16

**Authors:** Gao-Zhen Cao, Jia-Yi Huang, Qing-Shan Lin, Cong Chen, Min Wu, Run Wang, Ming-Yen Ng, Kai-Hang Yiu, Jian-Cheng Xiu

**Affiliations:** ^1^The First School of Clinical Medicine, Southern Medical University, 510000 Guangzhou, Guangdong, China; ^2^Division of Cardiology, Department of Medicine, The University of Hong Kong-Shenzhen Hospital, 518000 Shenzhen, Guangdong, China; ^3^Division of Cardiology, Department of Medicine, The University of Hong Kong, Queen Mary Hospital, Hong Kong, China; ^4^Division of Radiology, Department of Medicine, The University of Hong Kong-Shenzhen Hospital, 518000 Shenzhen, Guangdong, China

**Keywords:** cortisol, obesity, hypertension-mediated organ damage, untreated hypertension

## Abstract

**Background:**

Given the close relationship between excessive cortisol secretion and obesity, as well as their intimate associations with cardiometabolic sequelae, this study aimed to evaluate whether elevated cortisol levels and obesity are independently and potentially interactively related to hypertension-mediated organ damage (HMOD) in patients with untreated hypertension.

**Methods:**

A total of 936 untreated hypertensive patients were recruited. Body mass index (BMI), 24-hour urinary free cortisol (24 h UFC), and HMOD indicators, including left ventricular hypertrophy (LVH), carotid intima-media thickness (CIMT), and albuminuria, were assessed. Multivariate logistic regression was conducted to evaluate the associations of HMOD indicators with 24 h UFC and obesity. Generalized linear models were used to test for the interaction effects of obesity in the associations between log 24 h UFC levels and HMOD indicators.

**Results:**

Compared to non-obese patients, those who were obese had a greater left ventricular mass index (LVMI), greater CIMT, a higher level of 24-hour urinary albumin (24 h UALB) and more frequent albuminuria (all *p* < 0.05). In the obese group, elevated 24 h UFC was significantly associated with LVH (odds ratio (OR) = 2.53; 95% CI: 1.02–6.31, *p* = 0.044) and albuminuria (OR = 3.13; 95% CI: 1.31–7.43, *p* = 0.01), after multivariate adjusting. There was a significant interactive effect of obesity on the association between 24 h UFC and LVH and albuminuria (all *p* for interaction <0.05). A significant correlation was observed between 24 h UFC and LVMI in obese and non-obese patients. Conversely, the correlations of 24 h UFC and log 24 h UALB were found only in obese patients but not in non-obese patients.

**Conclusions:**

Elevated 24 h UFC levels were associated with higher severity of HMOD, including more frequent LVH, albuminuria, and greater CIMT. Additionally, obesity modified the effects of 24 h UFC on both LVH and albuminuria.

## 1. Introduction

The global prevalence of hypertension was estimated to be 1.13 billion in 2015; China alone experienced an increase to 244.5 million in 2015 [1]. Hypertension rarely occurs alone and may cause hypertension-mediated organ damage (HMOD), which refers to structural and functional changes in arteries or end organs, such as the heart, kidneys, and blood vessels. Mortality and morbidity are significantly worsened in hypertensive patients once HMOD occurs [2]. Thus, the current guidelines [3, 4, 5] recommend that all hypertensive patients undergo basic screening for HMOD, which may influence subsequent treatment decisions. As a result, understanding the factors contributing to the development of HMOD is clinically relevant in managing patients with hypertension.

Endogenous cortisol is a key hormone in regulating the immune system, glucose and lipid metabolism, water and electrolyte balance, blood pressure (BP), and heart rate (HR) maintenance. Excess cortisol secretion can lead to numerous negative health consequences involving multiple organs. A prior study has suggested that 24-hour urinary free cortisol (24 h UFC) or plasma cortisol is correlated with HMOD [6]. Our previous research also found more significant myocardial fibrosis and worse left ventricular dysfunction in Cushing’s syndrome patients exposed to higher cortisol levels than in essential hypertensive patients [7]. Obesity, another important risk factor for cardiovascular disease, was likewise shown to be closely related to HMOD [8, 9]. Rather than being merely independent factors, it has been hypothesized that a continuous loop may exist between obesity and increased cortisol that may worsen cardiometabolic sequelae [10]. While both are significant contributors, the potential interactive role of excess cortisol and obesity in the development of HMOD is unexplored. The present study aimed to evaluate whether elevated cortisol levels and obesity are independently and potentially interactively related to HMOD in patients with untreated hypertension.

## 2. Methods

### 2.1 Study Design and Study Population

**Fig. 1.**
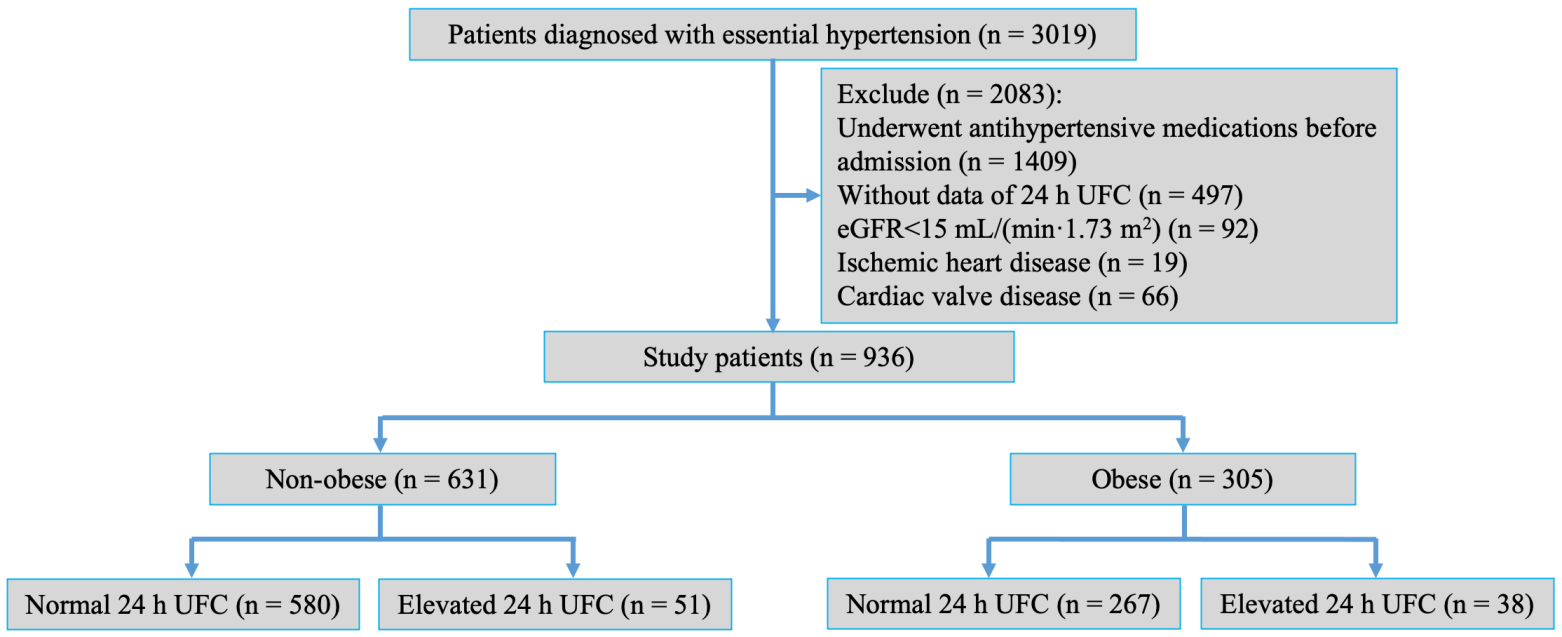
**A flow chart of the study**. Abbreviations: n, number; 24 h UFC, 24-hour urinary free cortisol; eGFR, estimated glomerular filtration rate.

This cross-sectional study was conducted at the Hypertension Center of the University of Hong Kong-Shen Zhen Hospital. Patients diagnosed with untreated essential hypertension were consecutively recruited from July 2016 to September 2021. The predefined exclusion criteria were age <18 years, secondary hypertension [3] and Cushing’s syndrome [11], use of corticosteroids, pregnancy or history of estrogen use, history of alcohol abuse, estimated glomerular filtration rate (eGFR) <15 mL/(min⋅1.73 m^2^), major depressive disorders, history of recent infection, ischemic heart disease, and cardiac valve disease. Patients with abnormal 24 h UFC underwent measurement of plasma cortisol levels at 8 AM, 4 PM, and 12 AM, followed by measurement of plasma cortisol levels after an overnight dexamethasone suppression test (DST) with 1 mg dexamethasone to exclude Cushing syndrome, according to guidelines [11]. All patients underwent either adrenal magnetic resonance imaging (MRI) or computed tomography (CT) imaging to exclude the presence of adrenal incidentalomas. Patients who administered any antihypertensive drug within 4 weeks before enrollment were further excluded (Fig. 1). Collected data included comprehensive medical history, body weight and height, and office BP measured according to the standard procedures in the current guidelines [3]; urine and blood samples and an echocardiogram. The study was conducted according to the principles of the Declaration of Helsinki and was approved by the local Institutional Review Board. Informed consent was obtained from all patients.

### 2.2 Definitions

Hypertension was defined as systolic blood pressure (SBP) ≥140 mmHg and/or diastolic blood pressure (DBP) ≥90 mmHg or a self-reported history of hypertension [3]. Obesity was defined as a body mass index (BMI) ≥28 kg/m^2^ [12] based on measured weight and height. Smokers were defined as those who smoked for ≥5 years and up to 1 year before enrollment. An elevated 24 h UFC was defined as 24 h UFC levels above the normal range (>403 µg/24 h, upper normal range value according to the current assay system). Albuminuria was defined as 24-hour urinary albumin (24 h UALB) ≥30 mg/24 h.

### 2.3 Office BP Measurement

Office BP measurements were performed according to the European Society of Hypertension and the European Society of Cardiology (ESH and ESC) recommendations [3] by a validated and calibrated BP measurement device (Omron HEM-7130; Omron Healthcare, Kyoto, Japan). Patients were seated comfortably in a quiet environment for at least 5 minutes before measurements. The arm for BP measurements was positioned on a desk at heart level. At two-minute intervals, measurements were initially taken from both arms, and then two additional measurements were taken from the arm with the highest initial reading. The average of the last two readings was documented for analysis purposes.

### 2.4 Laboratory Measurements

Assays for both 24 h UFC and 24 h UALB were performed using 24-hour urine following standardized procedures by the UniCel™ DxI 800 Access Immunoassay System (Beckman Coulter Inc., Brea, CA, USA). The blood sample was collected in the morning after at least 12 h of fasting and used for the biochemistry assays, including glycosylated hemoglobin (HbA1c), low-density lipoprotein cholesterol (LDL-C), serum creatinine (SCr), uric acid, and adrenocorticotropic hormone (ACTH), analyzed using a Roche COBAS 8000 device (Roche Diagnostic, Basel, Switzerland). The Modification of Diet in Renal Disease (MDRD) formula was used to calculate the eGFR [13].

### 2.5 Echocardiography

Standard 2-dimensional echocardiography and tissue Doppler imaging were performed on recruited patients with a commercially available echocardiography system (VingmedE9, General Electric Vingmed Ultrasound, Horten, Norway) by skilled operators who were masked to the clinical and biochemical characteristics of the patients. Patients were in the lateral decubitus position, and a 3.5 MHz transducer was used to capture images and digitally store them in cine-loop format. Left ventricular ejection fraction (LVEF) was determined from apical 4- and 2-chamber views using the modified Simpson’s biplane method. Left ventricular end-diastolic diameter (LVEDd) and left ventricular end-systolic diameter (LVESd), end-diastole interventricular septal thickness (IVSd), and posterior wall thickness (PWTd) were measured using the leading-edge-to-leading-edge method from 2-dimensional guided M-mode tracings recorded at the parasternal long-axis view [14]. Left ventricular mass index (LVMI) was estimated using the corrected American Society of Echocardiography (ASE) formula [14] (0.8 × (1.04 × ((IVSd + LVEDd + PWTd)^3^ – (LVEDd)^3^)) + 0.6) and was normalized to body surface area (BSA). Left ventricular hypertrophy (LVH) was defined as increased LVMI (≥95 g/m^2^ in females; ≥115 g/m^2^ in males). The ASE formula calculated relative wall thickness (RWT): RWT = 2 × PWTd/LVEDd. Increased RWT was defined as RWT >0.42 [14]. Left ventricular (LV) structural patterns were defined as normal (normal LVMI and normal RWT), concentric remodeling (normal LVMI and increased RWT), concentric hypertrophy (LVH and increased RWT), and eccentric hypertrophy (LVH and normal RWT). LV diastolic function was defined according to the ASE guideline (E-wave transmitral velocity to early diastolic velocity at tissue-Doppler imaging [E/e^′^] ratio >14 or meeting ≥2 of the following criteria: early-wave transmitral diastolic velocity/late-wave transmitral diastolic velocity ratio <1.0; left atrial volume >34 mL; early diastolic velocity of septal and lateral myocardial portions at tissue-Doppler imaging [e^′^] velocity <9 cm/s) [15]. Carotid intima-media thickness (CIMT) was defined as the mean value of the maximum far wall intima-media thickness of the right and left common carotid arteries measured by carotid ultrasound.

### 2.6 Statistical Analysis

The demographic and clinical characteristics of the enrolled study population were examined by obesity and 24 h UFC status. The Kolmogorov‒Smirnov test was used to assess the distribution of continuous variables. Normally distributed variables are presented as the mean ± standard deviation (SD). Non-normally distributed variables are presented as the median (interquartile range (IQR)) and were log-transformed to achieve a normal distribution before statistical testing. Categorical data are presented as the absolute number (percentage). Differences between groups were tested by Student’s *t*-test, the Mann‒Whitney test for continuous variables, and the χ^2^ test for categorical variables. Comparisons between groups (Group 1: non-obese with normal 24 h UFC, Group 2: non-obese with elevated 24 h UFC, Group 3: obese with normal 24 h UFC, Group 4: obese with elevated 24 h UFC) were conducted by analysis of variance (ANOVA) or the Bonferroni test for multiple comparisons. Multivariate logistic regression was performed to evaluate the associations between HMOD and 24 h UFC. To assess the predictive performance of 24 h UFC, logistic regression models were fitted with 24 h UFC alone and combined with other risk factors, comparing model fit using Nagelkerke R^2^. Generalized linear models examined the interaction effects of obesity on associations between log-transformed 24 h UFC levels and various HMOD indicators. Interaction plots illustrated associations between 24 h UFC levels and HMOD indicators across obesity and non-obesity groups. Statistical analyses were conducted using SPSS 21.0 (SPSS Inc, Chicago, IL, USA) and R software version 4.0.0 (The R Foundation, Vienna, Austria), with statistical significance set at *p* < 0.05 (two-tailed).

## 3. Results

### 3.1 Clinical Characteristics by 24 h UFC and Obesity Status

This study included 936 patients with essential hypertension (mean age 39.3 ± 8.9 years; 71% male). Among these, 305 patients (32.5%) were considered obese, and 89 patients (9.5%) had elevated 24 h UFC levels according to the predefined cutoff value. Obese patients exhibited higher 24-hour UFC levels compared to non-obese patients, while the ACTH levels were similar (**Supplementary Table 1**). Patients were further divided into four groups: (1) non-obese with normal 24 h UFC (n = 580, 62.0%); (2) non-obese with elevated 24 h UFC (n = 51, 5.4%); (3) obese with normal 24 h UFC (n = 267, 28.5%); (4) obese with elevated 24 h UFC (n = 38, 4.1%). Among non-obese patients, those with elevated 24 h UFC were more likely to be male and possess a higher prevalence of SBP, DBP, and diabetes compared to those with normal 24 h UFC. In obese patients, those with elevated 24 h UFC had higher SBP, DBP, HR, and lower eGFR compared to those with normal 24 h UFC. Additionally, these patients had a higher proportion of heart failure and statin use (Table 1).

**Table 1.** **Clinical characteristics according to 24 h UFC and obese status**.

		Non-obese (n = 631)			Obese (n = 305)		
		Normal 24 h UFC	Elevated 24 h UFC	*p*	Normal 24 h UFC	Elevated 24 h UFC	*p*
		(n = 580)	(n = 51)		(n = 267)	(n = 38)	
Demographic data							
	Age, years	40.2 ± 9.3	41.8 ± 9.2	0.636	37.2 ± 7.9	35.6 ± 5.7	0.696
	Male, n (%)	366 (63.1)	43 (84.3)	0.002	221 (82.8)	36 (94.7)	0.059
	BMI	24.3 ± 2.4	25.1 ± 2.0	0.232	31.4 ± 3.9	31.5 ± 3.0	0.997
	Smoking, n (%)	118 (20.3)	8 (15.7)	0.583	99 (37.1)	19 (50.0)	0.154
	Duration of hypertension, months	12.0 (2.0–36.0)	24.0 (6.0–60.0)	0.298	18.0 (1.0–36.0)	18.0 (3.0–48.0)	0.935
	SBP, mmHg	157.8 ± 25.3	174.4 ± 31.9	<0.001	161.5 ± 25.2	194.4 ± 29.2	<0.001
	DBP, mmHg	101.4 ± 17.3	110.5 ± 23.1	0.003	105.4 ± 17.5	128.3 ± 21.3	<0.001
	HR, bpm	83.6 ± 13.5	87.1 ± 14.5	0.305	86.2 ± 14.0	101.0 ± 14.5	<0.001
Medical and drug history							
	AF, n (%)	4 (0.7)	1 (2.0)	0.345	2 (0.7)	0 (0)	1.000
	Diabetes, n (%)	75 (12.9)	13 (25.5)	0.019	63 (23.6)	8 (21.1)	0.839
	HF, n (%)	7 (1.2)	1 (2.0)	0.492	10 (3.7)	5 (13.2)	0.027
	Stroke, n (%)	11 (1.9)	0 (0)	0.393	7 (2.6)	2 (5.3)	0.311
	Statin use, n (%)	80 (13.8)	8 (15.7)	0.833	43 (16.1)	12 (31.6)	0.026
Biochemical variables							
	24 h UFC, µg/24 h	190.5 (139.0–251.0)	482.0 (440.0–612.0)	0.000	223.0 (167.0–284.0)	666.0 (501.3–861.8)	0.000
	ACTH, pg/mL	18.4 (10.8–28.7)	21.1 (12.3–35.4)	0.114	20.8 (13.4–33.7)	20.8 (14.3–43.8)	0.995
	eGFR, mL/min/1.73 m^2^	101.4 ± 26.1	91.8 ± 26.2	0.077	101.9 ± 30.0	83.7 ± 31.2	0.001
	HbA1c, %	5.6 ± 0.6	5.8 ± 1.0	0.293	5.9 ± 1.1	5.8 ± 1.2	0.843
	LDL, mmol/L	3.1 ± 0.9	3.2 ± 1.0	0.576	3.3 ± 1.0	3.4 ± 0.7	1.000
	Uric acid, µmol/L	376.8 ± 104.3	397.5 ± 108.5	0.518	450.0 ± 99.7	465.8 ± 98.6	0.813

Abbreviations: n, number; 24 h UFC, 24-hour urinary free cortisol; BMI, body mass index; SBP, systolic blood pressure; DBP, diastolic blood pressure; HR, heart rate; AF, atrial fibrillation; HF, heart failure; ACTH, adrenocorticotropic hormone; eGFR, estimated glomerular filtration rate; HbA1c, glycosylated hemoglobin; LDL, low-density lipoprotein.

### 3.2 HMOD Indicators According to 24 h UFC and Obesity Status

**Fig. 2.**
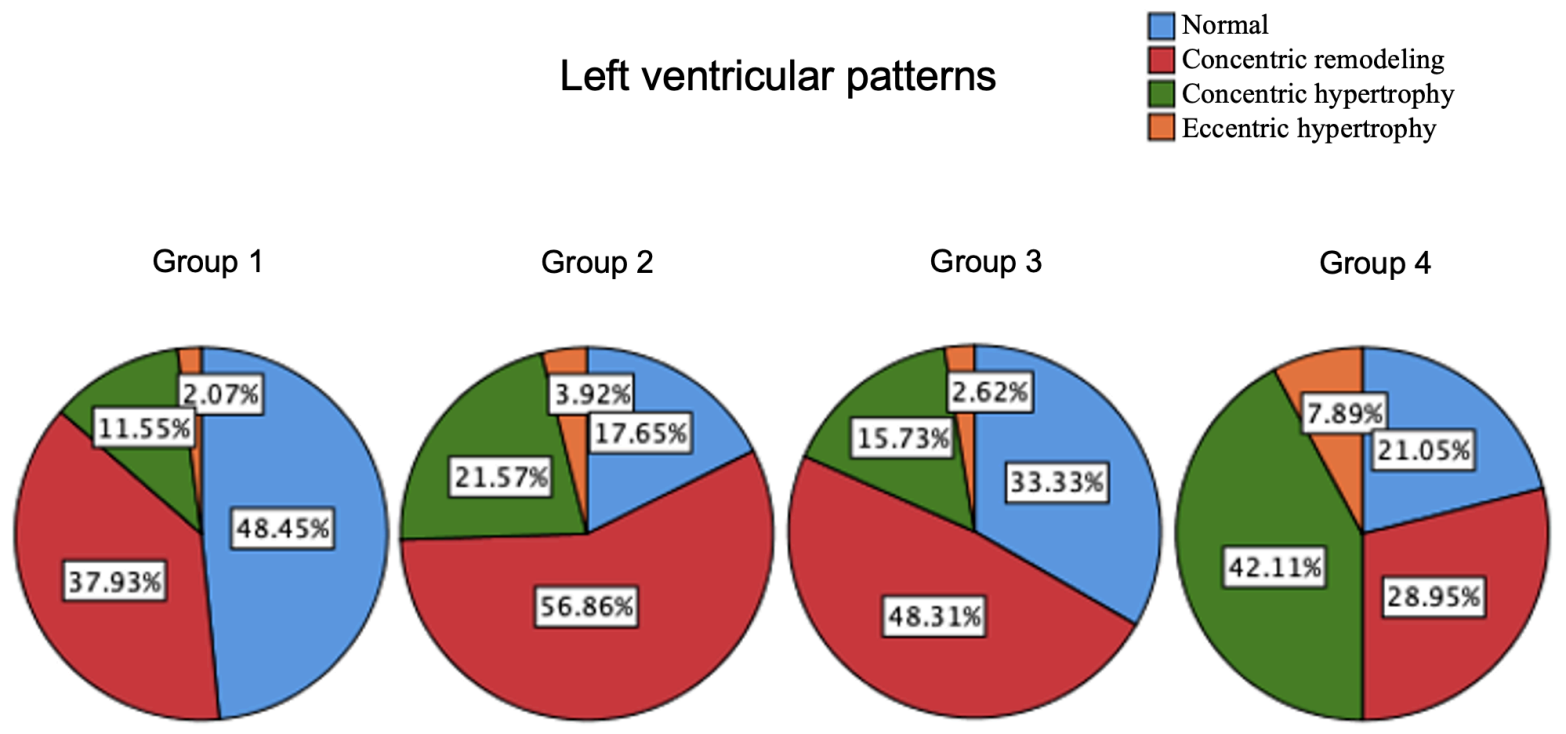
**Distribution of various patterns of left ventricular hypertrophy in the four groups**. Group 1: Non-obesity with normal 24 h UFC; Group 2: Non-obesity with elevated 24 h UFC; Group 3: Obesity with normal 24 h UFC; Group 4: Obesity with elevated 24 h UFC. Abbreviations: 24 h UFC, 24-hour urinary free cortisol.

Compared to non-obese patients, obese patients had higher LVMI, greater CIMT, and more frequent albuminuria (all *p* < 0.05). Similarly, patients with elevated 24 h UFC exhibited characteristics similar to those with normal 24 h UFC (**Supplementary Table 2**). Among non-obese patients, those with elevated 24 h UFC had higher LVMI, CIMT, and 24 h UALB levels than those with normal 24 h UFC. In obese patients, those with elevated 24 h UFC had higher LVMI, increased 24 h UALB, and lower LVEF compared to those with normal 24 h UFC. There were no significant differences in CIMT and carotid plaques (Table 2). The proportions of LVH geometry in the four groups were significantly different (Fig. 2), with obese patients with elevated 24 h UFC having the highest percentages of concentric (42.1%) and eccentric (7.9%) LVH.

**Table 2.** **Echocardiographic variables and HMOD indicators according to 24 h UFC and obese status**.

	Non-obese (n = 631)			Obese (n = 305)		
	Normal 24 h UFC	Elevated 24 h UFC	*p*	Normal 24 h UFC	Elevated 24 h UFC	*p*
	(n = 580)	(n = 51)		(n = 267)	(n = 38)	
LAVI, mL/m^2^	11.6 ± 4.0	13.7 ± 4.8	<0.001	13.8 ± 6.0	19.4 ± 11.7	<0.001
LVEDV, mL	99.0 ± 23.1	102.3 ± 21.8	0.329	113.3 ± 30.5	140.0 ± 49.3	<0.001
LVESV, mL	33.8 ± 13.1	35.0 ± 11.0	0.533	40.7 ± 21.3	61.4 ± 40.8	<0.001
RWT	0.43 ± 0.07	0.47 ± 0.8	<0.001	0.45 ± 0.08	0.47 ± 0.08	0.118
LVMI, g/m^2^	86.3 ± 25.6	98.3 ± 25.1	0.001	93.2 ± 25.3	128.0 ± 52.1	<0.001
LVEF, %	66.3 ± 6.0	66.1 ± 5.2	0.783	64.8 ± 7.1	59.4 ± 12.2	<0.001
LVEF <50%, n (%)	7 (1.2)	0 (0)	0.090	8 (3.0)	7 (18.4)	<0.001
E/A ratio	1.1 ± 0.3	1.0 ± 0.4	0.091	1.1 ± 0.4	1.0 ± 0.4	0.268
E/e′ ratio	9.5 ± 3.2	9.9 ± 3.6	0.450	10.2 ± 3.5	12.3 ± 3.7	0.001
24 h UALB, mg/24 h	9.1 (4.6–28.9)	22.3 (6.3–44.2)	0.017	18.4 (7.9–57.0)	92.8 (24.4–451.5)	<0.001
Albuminuria, n (%)	148 (25.5)	15 (29.4)	0.308	99 (37.1)	26 (68.4)	<0.001
CIMT, mm	0.81 ± 0.20	0.91 ± 0.23	0.012	0.85 ± 0.20	0.90 ± 0.18	0.337
Carotid plaque, n (%)	122 (21.0)	13 (25.5)	0.412	53 (19.9)	11 (28.9)	0.205

Abbreviations: HMOD, hypertension-mediated organ damage; n, number; 24 h UFC, 24-hour urinary free cortisol; LAVI, left atrial volume index; LVEDV, left ventricular end-diastolic volume; LVESV, left ventricular end-systolic volume; RWT, relative wall thickness; LVMI, left ventricular mass index; LVEF, left ventricular ejection fraction; E/A, early wave transmitral diastolic velocity/late-wave transmitral diastolic velocity; E/e^′^, E-wave transmitral velocity to early diastolic velocity at tissue Doppler imaging; UALB, urinary albumin; CIMT, carotid intimal medial thickness.

### 3.3 Effect of Obesity on the Relationship between 24 h UFC and HMOD

**Fig. 3.**
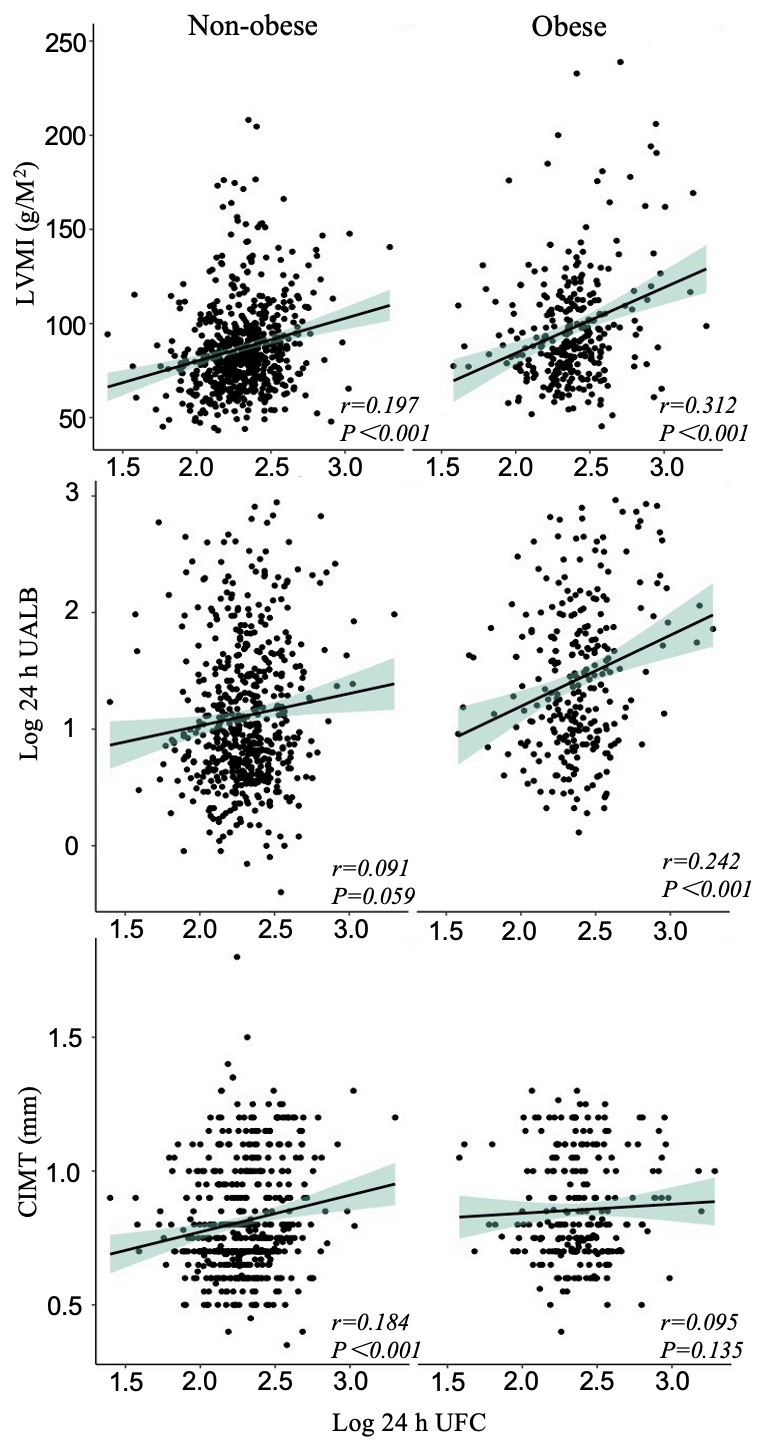
**The correlations between 24-hour UFC and HMOD indicators in non-obese and obese patients**. Abbreviations: 24 h UFC, 24-hour urinary free cortisol; HMOD, hypertension-mediated organ damage; LVMI, left ventricular mass index; UALB, urinary albumin; CIMT, carotid intimal medial thickness.

The prevalence of LVH was 22.3% in the obese group, while the prevalence of albuminuria and carotid plaque were 41% and 21%, respectively (Table 3). After multivariate adjusting for age, sex, smoking, duration of hypertension, diabetes, SBP, DBP, HR, and eGFR, elevated 24 h UFC was significantly associated with LVH (odds ratio (OR) = 2.53; 95% CI: 1.02–6.31, *p* = 0.044) and albuminuria (OR = 3.13; 95% CI: 1.31–7.43, *p* = 0.01) in the obese group. There was a significant interactive effect of obesity on the association between 24 h UFC and LVH and albuminuria (all *p* for interaction <0.05) but not on carotid plaques. Fig. 3 demonstrates how obesity modifies the relationships between 24 h UFC and LVMI, log 24 h UALB, and CIMT. A significant correlation was observed between 24 h UFC and LVMI in obese and non-obese patients. Conversely, 24 h UFC and log 24 h UALB correlations were found only in obese patients, whereas a significant correlation between 24 h UFC and CIMT was found only in non-obese.

**Table 3.** **Multivariate logistic regression of HMOD indicators and 24 h UFC in obese and non-obese patients**.

		Numbers of event (%)	Normal 24 h UFC	Elevated 24 h UFC						*p *_interaction_
				Model 1		Model 2		Model 3		
				OR (95% CI)	Nagelkerke R^2^	OR (95% CI)	Nagelkerke R^2^	OR (95% CI)	Nagelkerke R^2^	
LVH	Non-obese	95 (15.1%)	Ref	2.08 (1.06–4.07)*	0.005	2.05 (1.03–4.09)*	0.047	1.45 (0.65–3.20)	0.239	0.046
	Obese	68 (22.3%)	Ref	4.45 (2.19–9.03)*	0.072	5.21 (2.51–10.83)*	0.089	2.53 (1.02–6.31)*	0.316	
Albuminuria	Non-obese	163 (25.8%)	Ref	1.40 (0.73–2.70)	0.001	1.49 (0.77–2.90)	0.015	1.25 (0.63–2.51)	0.043	0.010
	Obese	125 (41.0%)	Ref	3.89 (1.80–8.42)*	0.052	3.97 (1.82–8.63)*	0.053	3.13 (1.31–7.43)*	0.072	
Carotid plaque	Non-obese	135 (21.4%)	Ref	1.32 (0.68–2.57)	0.001	1.05 (0.51–2.16)	0.188	0.92 (0.43–1.97)	0.202	0.190
	Obese	64 (21.0%)	Ref	1.64 (0.76–3.52)	0.006	1.88 (0.84–4.17)	0.138	1.78 (0.70–4.54)	0.164	

Abbreviations: HMOD, hypertension-mediated organ damage; 24 h UFC, 24-hour urinary free cortisol; OR odds ratio; CI, confidence interval; LVH, left ventricular hypertrophy; Ref, reference; *, *p* < 0.005; Model 1, unadjusted model; Model 2, adjusted for age and sex; Model 3, adjusted for age, sex, smoking, duration of hypertension, diabetes, SBP, DBP, HR, and eGFR. SBP, systolic blood pressure; DBP, diastolic blood pressure; HR, heart rate; eGFR, estimated glomerular filtration rate.

## 4. Discussion

The present study shows that elevated 24-hour UFC levels and obesity are related to adverse LV remodeling, albuminuria, and increased CIMT in patients with untreated hypertension. A significant interaction was observed between 24 h UFC and obesity in relation to LVH and albuminuria, but not carotid plaques. Our findings highlight the potential role of 24-hour UFC levels in the pathogenesis of HMOD, especially for obese patients.

Even without Cushing’s syndrome, elevated cortisol levels are common in hypertensive patients [6]. Our study found elevated 24 h UFC levels in 10.5% of untreated hypertensive patients. Beyond the link between cortisol and high blood pressure, increased cortisol levels contribute to cardiovascular abnormalities. Studies consistently show that both Cushing’s syndrome and isolated elevated cortisol are associated with adverse LV remodeling and myocardial dysfunction [16, 17]. A meta-analysis confirmed the correlation between Cushing’s syndrome and CIMT [18]. A recent case–control study indicated that hair cortisol concentration significantly predicts coronary atherosclerosis [19], while another cohort study in older individuals found that salivary cortisol is related to carotid artery atherosclerosis [20]. Additionally, a survey of patients with type 2 diabetes and prediabetes demonstrated a correlation between high serum cortisol and microalbuminuria [21]. Although it is debated whether elevated cortisol levels contribute to HMOD, our results show that excess cortisol correlates with HMOD, including adverse LV remodeling, CIMT, and albuminuria in untreated hypertensive patients. These findings highlight the need for further research into antihypertensive treatments targeting cortisol excess.

Obesity has been regarded as one of the critical risk factors for cardiovascular complications [22, 23], and the prevalence is increasing globally, reaching 19.5% in 2015 [24]. In our present cohort, the prevalence of obesity reached 32.5%, which is higher than the figures from previous studies involving patients with hypertension, ranging from 13 to 20% [25, 26, 27]. A recent community-based study demonstrated that body measurements, including BMI and waist–hip ratio, were closely related to HMOD in an older population [9]. A recent national health survey reported that CIMT is similarly associated with obesity and hypertension in young patients [28]. In a study that involved 2350 subjects aged 40 years or older, obesity was also shown to be related to albuminuria [29]. Our study further validated the close relationship between HMOD and obesity by assessing the degree of LVH, impaired LVEF, diastolic function, CIMT, and albuminuria in a cohort of untreated hypertensive patients. The importance of weight reduction has been well verified in prior studies and should be further evaluated and reinforced, particularly for hypertensive patients at risk of HMOD.

While both excess cortisol and obesity are independently associated with HMOD, whether an interaction exists between the two in LV remodeling, atherosclerosis, and albuminuria is uncertain. Given the frequent prevalence of these two factors in patients with hypertension, their potential interdependent role in relation to HMOD deserves clarification. Our study demonstrates for the first time that untreated hypertension patients with excess cortisol and obesity exhibit a pronounced worsening of LV adverse remodeling and albuminuria. The interactive association suggests that excess cortisol and obesity may provide a unique and expanded relation to key HMOD cases, further translating into clinical consequences. Several reasons may explain the interaction between excess cortisol and obesity regarding HMOD: Firstly, beyond hypertension, chronic exposure to high cortisol levels is linked to other metabolic abnormalities, such as central obesity, insulin resistance, hyperglycemia, and dyslipidemia [30]. The obese phenotype may reflect these cortisol-induced metabolic abnormalities, which are closely associated with HMOD. Secondly, the effects of cortisol on tissues and organs are regulated by 11β-hydroxysteroid dehydrogenase type 1 (HSD1) and type 2 (HSD2); 11β-HSD1 converts inactive cortisone to active cortisol [31, 32], thereby increasing circulating and local cortisol concentrations. Conversely, 11β-HSD2 inactivates cortisol [33]. Although data are inconsistent, evidence suggests that in obese individuals, the upregulated activity of 11β-HSD in visceral and hepatic tissues may amplify the local effects of glucocorticoids [34]. The assessment of cortisol levels and body measurements may enable clinicians to distinguish those who may experience HMOD, which merits detailed assessment and frequent surveillance for complications.

Interestingly, our study revealed that the correlation between CIMT and 24 h UFC was significantly positive only in the non-obese group, while not significant in the obese group. We speculate that this may be attributed to two possible factors. Firstly, the elevated 24 h UFC group in the obese population featured a larger proportion of statin use in comparison to the normal 24 h UFC group (31.6% *vs*. 16.1%, *p* = 0.026). As such, the use of statin, a known modifier of atherosclerosis, may attenuate the impact of heightened cortisol levels on CIMT among obese patients [35]. Secondly, the elevated 24 h UFC group in the obese demographic showcased fewer cases (n = 38), which may have limited the demonstration of a significant association between CIMT and cortisol levels. Future studies with a greater number of cases and that consider statin therapy are needed to clarify the relationship between CIMT and 24-hour UFC levels in obese patients.

## 5. Strengths and Limitations

While most studies that evaluate HMOD involve both treated and untreated hypertensive patients, our cohort included only those who had not been treated previously. This could minimize the potential modifying effects of antihypertensive drugs on HMOD, which may invalidate our observed correlations. Furthermore, as cortisol exhibits a circadian rhythm, we measured 24-hour UFC levels, which could better represent the daily excess cortisol exposure. Finally, our study included a large group of untreated hypertensive patients, which enabled us to capture enough HMOD outcome data for robust statistical analysis.

Our present study has several limitations. A causal relationship between HMOD and elevated cortisol levels and obesity cannot be established due to the cross-sectional design. Assessing cortisol exposure through a single measurement of 24 h UFC may not be entirely reliable due to daily variations in 24 h UFC levels [36]. Overnight DST plasma cortisol levels were measured only in patients with abnormal 24 h UFC, potentially overlooking the existence of subclinical Cushing’s syndrome within the study’s participant population. Additionally, this study lacks data from normotensive subjects as a separate control group. While CIMT is an established marker for subclinical atherosclerosis, other modalities, such as coronary calcification, should also be considered in future studies. Although albuminuria is an established marker of renal involvement, the use of additional sensitive markers, such as microalbuminuria or cystatin C, in patients with hypertension could verify our current results.

## 6. Conclusions

In a large cohort of untreated hypertensive patients, we observed thatelevated 24 h UFC levels were associated with higher HMOD severity, including more frequent LVH, albuminuria, and greater CIMT. Additionally, obesity modified the effects of 24 h UFC on both LVH and albuminuria. Future studies are encouraged to explore methods for reducing excess cortisol, especially in obese patients, to prevent the potential development of HMOD.

## Availability of Data and Materials

We are unable to disclose the complete set of original data, as it comprises a significant amount of confidential information pertaining to our patients.

## Supplementary Material

Supplementary material associated with this article can be found, in the online version, at https://doi.org/10.31083/RCM25598.
