# Supplementary material for: Interaction between 24 h Urinary Free Cortisol and Obesity in Hypertension-Mediated Organ Damage in Patients with Untreated Hypertension
Source: Rev Cardiovasc Med. 2025 Jan 16;26(1):25598. doi: 10.31083/RCM25598 (PMC11760541; doi:10.31083/RCM25598)
Supplement: Supplementary file 1 [file 2153-8174-26-1-25598-s1.doc]

**Supplementary Appendix**

**Supplementary Table 1. Baseline characteristics by obese and 24-UFC status, respectively.**

|  | Nonobese  (n=631) | Obese  (n=305) | P | Normal 24h-UFC (n=847) | Elevated 24h-UFC (n=89) | P |
| --- | --- | --- | --- | --- | --- | --- |
| **Demographic data** |  |  |  |  |  |  |
| Age, years | 40.4±9.3 | 37.0±7.6 | <0.001 | 39.3±8.9 | 39.1±8.5 | 0.862 |
| Male, n (%) | 409 (64.8) | 257 (84.3) | <0.001 | 587 (69.3) | 79 (88.8) | <0.001 |
| BMI | 24.3±2.4 | 31.4±3.8 | <0.001 | 26.5±4.5 | 27.8±4.0 | 0.005 |
| Smoking, n (%) | 126 (20.0) | 118 (38.7) | <0.001 | 217 (25.6) | 27 (30.3) | 0.200 |
| Duration of hypertension, months | 12.0 (2.0~36.0) | 18.0 (1.0~36.0) | 0.783 | 12.0 (2.0~36.0) | 24.0 (5.0~48.0) | 0.158 |
| SBP, mmHg | 159.1±26.3 | 165.6±27.9 | 0.001 | 159.0±25.4 | 182.9±42.2 | <0.001 |
| DBP, mmHg | 102.1±18.0 | 108.3±19.5 | <0.001 | 102.6±17.4 | 118.1±24.0 | <0.001 |
| HR, bpm | 83.9±13.6 | 88.1±14.9 | <0.001 | 84.5±13.7 | 93.0±14.0 | <0.001 |
| **Medical and drug history** |  |  |  |  |  |  |
| AF, n (%) | 5 (0.8) | 2 (0.7) | 0.588 | 6 (0.7) | 1 (1.1) | 0.504 |
| Diabetes, n (%) | 88 (13.9) | 71 (23.3) | <0.001 | 138 (16.3) | 21 (23.6) | 0.059 |
| HF, n (%) | 8 (1.3) | 15 (4.9) | 0.001 | 17 (2.0) | 6 (6.7) | 0.017 |
| Stroke, n (%) | 11 (1.7) | 9 (3.0) | 0.169 | 18 (2.1) | 2 (2.2) | 0.582 |
| Statin use (%) | 88 (13.9) | 55 (18.0) | 0.121 | 123 (14.5) | 20 (22.5) | 0.062 |
| **Biochemical variables** |  |  |  |  |  |  |
| 24h-UFC, ug/24h | 203.0 (143.0~278.0) | 233.0 (174.0~324.0) | 0.000 | 203.0 (146.0~265.0) | 546.0 (459.0~725.0) | 0.000 |
| ACTH, pg/mL | 19.0 (11.0~29.0) | 20.8 (13.6~35.9) | 0.058 | 19.4 (11.7~29.6) | 21.1 (14.3~41.8) | 0.012 |
| eGFR, mL/min/1.73m^2^ | 100.6±26.2 | 99.6±30.7 | 0.618 | 101.6±27.4 | 88.3±28.5 | <0.001 |
| HbA1c, % | 5.6±0.6 | 5.9±1.1 | <0.001 | 5.7±0.8 | 5.8±1.0 | 0.303 |
| LDL, mmol/L | 3.1±0.9 | 3.3±0.9 | <0.001 | 3.1±0.9 | 3.3±0.8 | 0.159 |
| Uric acid, umol/L | 378.5±104.7 | 452.0±99.5 | <0.001 | 400.0±108.3 | 426.6±109.2 | 0.031 |

**Abbreviations:** n, number; UFC, urinary free cortisol; BMI, body mass index; SBP, systolic blood pressure; DBP, diastolic blood pressure; HR, heart rate; AF, atrial fibrillation; HF heart failure; ACTH, adrenocorticotropic hormone; eGFR, estimated glomerular filtration rate; HbA1c, glycosylated hemoglobin; LDL, low-density lipoprotein.

**Supplementary Table 2. Baseline HMOD indicators by obese and 24-UFC status.**

|  | Nonobese  (n=631) | Obese  (n=305) | P | Normal 24h-UFC (n=847) | Elevated 24h-UFC (n=89) | P |
| --- | --- | --- | --- | --- | --- | --- |
| LAVI, ml/m^2^ | 11.8±4.1 | 14.5±7.2 | <0.001 | 12.3±4.8 | 16.9±8.8 | <0.001 |
| LVEDV, mL | 99.3±23.0 | 116.7±34.4 | <0.001 | 103.5±26.5 | 118.2±40.4 | <0.001 |
| LVESV, mL | 33.9±12.9 | 43.2±25.4 | <0.001 | 35.9±16.4 | 46.1±30.5 | <0.001 |
| RWT | 0.43±0.07 | 0.48±0.08 | <0.001 | 0.43±0.07 | 0.47±0.08 | <0.001 |
| LVMI, g/m^2^ | 87.3±25.8 | 97.5±32.0 | <0.001 | 88.5±25.7 | 111.0±41.5 | <0.0001 |
| LVEF, % | 66.3±5.9 | 64.1±8.1 | <0.001 | 65.8±6.4 | 63.2±9.5 | 0.001 |
| LVEF<50%, n (%) | 7 (1.1) | 15 (4.9) | 0.001 | 15 (1.8) | 7 (7.9) | 0.003 |
| E/A ratio | 1.1±0.3 | 1.1±0.4 | 0.326 | 1.1±0.4 | 1.0±0.4 | 0.037 |
| E/e' ratio | 9.5±3.2 | 10.5±3.6 | <0.001 | 9.7±3.3 | 10.9±3.9 | 0.001 |
| 24h-UALB, mg/24h | 9.4 (4.7~30.0) | 22.2 (8.5~70.0) | <0.001 | 11.2 (5.3~38.1) | 33.7 (9.4~174.2) | <0.001 |
| Albuminuria, n (%) | 163 (28.2) | 125 (41.0) | <0.001 | 247 (29.2) | 41 (46.1) | <0.001 |
| CIMT, mm | 0.81±0.21 | 0.86±0.19 | 0.008 | 0.82±0.20 | 0.91±0.21 | <0.001 |
| Carotid plaque, n (%) | 135 (21.4) | 64 (21.0) | 0.511 | 175 (20.7) | 24 (27.0) | 0.151 |

**Abbreviations:** HMOD, hypertension-mediated organ damage; n, number; UFC, urinary free cortisol; LAVI, left atrial volume index; LVEDV, left ventricular end-diastolic volume; LVESV, left ventricular end-systolic volume; RWT, relative wall thickness; LVMI, left ventricular mass index; LVEF, left ventricular ejection fraction; E/A, early wave transmitral diastolic velocity/late-wave transmitral diastolic velocity; E/e′, E-wave transmitral velocity to early diastolic velocity at tissue-Doppler imaging; UALB, urinary albumin; CIMT, carotid intimal medial thickness.
